# Supplementary material for: Low COVID-19 Vaccine Acceptance Is Correlated with Conspiracy Beliefs among University Students in Jordan
Source: Int J Environ Res Public Health. 2021 Mar 1;18(5):2407. doi: 10.3390/ijerph18052407 (PMC7967761; doi:10.3390/ijerph18052407)
Supplement: Supplementary file 1 [file ijerph-18-02407-s001.pdf]

Supplementary S1

Consent form and questionnaire translated to English (the original form in Arabic is provided below).

Acceptance of COVID-19 Vaccination among University Students in Jordan

This questionnaire has been prepared to measure the extent of COVID-19 vaccination acceptance among university students in Jordan. The information provided through this questionnaire will be used for research purposes only, and the data will be treated with complete confidentiality and privacy.

Participation in this survey is completely voluntary.

The average time to complete the questionnaire is only 3 minutes.

Thank you very much for agreeing to take part in this survey.

Please confirm your consent to participate in the study:

☐ I agree

1. Age

………………….

2. Sex

☐ Male

☐ Female

3. Nationality:

………………….

4. University:

………………….

5. School/Faculty:

………………….

6. Educational level

☐ Undergraduate

☐ Postgraduate

7. Do you suffer from any chronic diseases (such as diabetes, allergy, hypertension or heart disease)?

☐ Yes

☐ No

8. Have you or any of your family members had COVID-19?

☐ Yes

☐ No

9. What is your belief about the origin of the current coronavirus in humans?

☐ Natural source from animals

☐ Man-made virus and part of a conspiracy plot

☐ No opinion

10. Do you think the current coronavirus was man-made to force everyone to get vaccinated?

☐ Yes

☐ No

☐ Maybe

11. Did you get COVID-19 vaccine/Do you intend to get COVID-19 vaccine?

- Yes
- No
- Maybe

12. Did you get influenza vaccine/Do you intend to get influenza vaccine?

- Yes
- No
- Maybe

13. Do you oppose vaccination altogether?

- Yes
- No
- No opinion

14. Do you think that COVID-19 vaccine will be a way of implanting people with microchips in order to control humans?

- Yes
- No
- Maybe

15. Do you think that COVID-19 vaccines will lead to infertility?

- Yes
- No
- Maybe

16. What is your main source of information about coronavirus vaccine?

- TV programs and news releases
- Social media platforms (Facebook, Twitter, Instagram, WhatsApp)/YouTube
- Medical doctors, scientists and scientific journals

For each statement, please indicate how much you disagree or agree by selecting the appropriate number:

**Item**	*Strongly Disagree*	*Disagree*	*Somewhat Disagree*	*Neutral*	*Somewhat Agree*	*Agree*	*Strongly Agree*
17. COVID-19 vaccine safety data is often fabricated.	☐	☐	☐	☐	☐	☐	☐
18. People are deceived about COVID-19 vaccine efficacy.	☐	☐	☐	☐	☐	☐	☐
19. Pharmaceutical companies cover up the dangers of COVID-19 vaccines.	☐	☐	☐	☐	☐	☐	☐
20. Vaccine efficacy data is often fabricated.	☐	☐	☐	☐	☐	☐	☐
21. People are deceived about vaccine safety.	☐	☐	☐	☐	☐	☐	☐
22. Immunizing children is harmful, and this fact is covered up.	☐	☐	☐	☐	☐	☐	☐
23. The government is trying to cover up the link between vaccines and autism.	☐	☐	☐	☐	☐	☐	☐

Thank you very much for participating in the survey

مستويات قبول لقاح كوفيد-١٩ لدى طلاب الجامعات في الأردن

تم إعداد هذا الاستبيان لقياس مستويات قبول لقاح كوفيد-١٩ لدى طلاب الجامعات في الأردن . سيتم استخدام المعلومات المقدمة لك من خلال هذا الاستبيان لأغراض بحثية فقط وسيتم التعامل مع البيانات بسرية وخصوصية تامة

المشاركة في هذا الاستبيان طوعية تمامًا

معدل وقت استكمال الاستبيان هو ٣ دقائق فقط

شكراً جزيلاً للموافقة على المشاركة في هذا الاستبيان

الرجاء تأكيد موافقتك على المشاركة في الدراسة

☐ **أوافق**

العمر: ………

الجنس:

☐ **ذكر**

☐ **أنثى**

الجنسية:

………

الجامعة:

………

الكلية:

………

المستوى التعليمي:

- ☐ **دبلوم أو بكالوريوس**
- ☐ **ماجستير أو دكتوراه**

هل تعاني من أي أمراض مزمنة (مثل السكري أو الحساسية، أو الضغط، أو أمراض في القلب)؟

- ☐ **نعم**
- ☐ **لا**

هل أُصبت أنت أو أحد أفراد أسرتك بكوفيد ١٩؟

- ☐ **نعم**
- ☐ **لا**

ما هو اعتقادك بشأن مصدر فيروس كورونا في البشر؟

- ☐ **مصدر طبيعي من الحيوانات**
- ☐ **فيروس مُصنع وجزء من مؤامرة**
- ☐ **ليس عندي رأي**

هل تعتقد أن فيروس كورونا تم تصنيعه لإجبار الجميع على أخذ اللقاح؟

- ☐ **نعم**
- ☐ **لا**
- ☐ **ربما**

هل ستأخذ لقاح الكورونا عند توفره؟

- ☐ **نعم**
- ☐ **لا**
- ☐ **ربما**

هل أخذت أو ستأخذ لقاح الانفلونزا؟

- ☐ **نعم**
- ☐ **لا**
- ☐ **ربما**

هل أنت من معارضين اللقاحات إجمالاً؟

- ☐ **نعم**
- ☐ **لا**
- ☐ **ليس عندي رأي**

هل تعتقد أن لقاح كوفيد ١٩ سيكون وسيلة لحقن الناس بشرائح مجهرية للسيطرة على البشر؟

- ☐ **نعم**
- ☐ **لا**
- ☐ **ربما**

سيؤدي لقاح كوفيد ١٩ إلى العُقم

- ☐ **نعم**
- ☐ **لا**
- ☐ **ربما**

ما هو المصدر الرئيسي لمعلوماتك حول اللقاح؟

- ☐ **برامج التلفاز و النشرات الإخبارية**
- ☐ **وسائل التواصل الاجتماعي (فيسبوك، تويتر، إنستغرام، يوتيوب)**
- ☐ **الأطباء والعلماء والمجلات العلمية المتخصصة**

لكل عبارة ، يُرجى توضيح مدى عدم موافقتك أو موافقتك عن طريق تحديد الرقم المناسب:

	أرفض بشدة	أعارض	أعارض إلى حدٍ ما	مُحايد	أوافق إلى حدٍ ما	أوافق	أوافق بشدة
بيانات سلامة لقاح كوفيد مُزيفة	☐	☐	☐	☐	☐	☐	☐
يتم خداع الناس بشأن فعالية لقاح كوفيد ١٩	☐	☐	☐	☐	☐	☐	☐
تُخفي شركات الأدوية عن الناس مخاطر لقاح كوفيد ١٩	☐	☐	☐	☐	☐	☐	☐
غالبًا ما تكون بيانات فعالية اللقاحات مُزيفة	☐	☐	☐	☐	☐	☐	☐
يتم خداع الناس بشأن سلامة اللقاحات	☐	☐	☐	☐	☐	☐	☐
تطعيم الأطفال ضار وهذه الحقيقة محجوبة عن الناس	☐	☐	☐	☐	☐	☐	☐
تحاول الحكومة التستر على الصلة بين اللقاحات وأمراض أُخرى مثل التوحد	☐	☐	☐	☐	☐	☐	☐

شكراً جزيلاً على مُشاركتكم في الاستبيان
